# Elevated peripheral blood neutrophil-to-lymphocyte ratio is associated with an immunosuppressive tumour microenvironment and decreased benefit of PD-1 antibody in advanced gastric cancer

**DOI:** 10.1093/gastro/goab032

**Published:** 2021-10-05

**Authors:** Dan-Yun Ruan, Yan-Xing Chen, Xiao-Li Wei, Ying-Nan Wang, Zi-Xian Wang, Hao-Xiang Wu, Rui-Hua Xu, Shu-Qiang Yuan, Feng-Hua Wang

**Affiliations:** 1 State Key Laboratory of Oncology in South China, Collaborative Innovation Center for Cancer Medicine, Sun Yat-sen University Cancer Center, Guangzhou, Guangdong, P. R. China; 2 Department of Medical Oncology, the Third Affiliated Hospital of Sun Yat-sen University, Guangzhou, Guangdong, P. R. China; 3 Department of Medical Oncology, Sun Yat-sen University Cancer Center, Guangzhou, Guangdong, P. R. China; 4 Department of Gastric Surgery, Sun Yat-sen University Cancer Center, Guangzhou, Guangdong, P. R. China

**Keywords:** advanced gastric cancer, neutrophil-to-lymphocyte ratio, anti-PD-1 monoclonal antibody, tumour microenvironment

## Abstract

**Background:**

Due to its limited efficacy and potential toxicity, anti-PD-1 monoclonal antibody is not suitable for all advanced gastric cancer (AGC) patients and predictive biomarkers identifying patients who can benefit from it are urgently needed. This study aimed to evaluate the predictive and prognostic value of inflammatory markers in the context of the systemic inflammatory status and tumour microenvironment.

**Methods:**

The study included 58 patients from a prospective study investigating the safety and efficacy of toripalimab in chemorefractory AGC patients. Patient characteristics, treatment outcomes, and haematological parameters were analysed. Immune-cell infiltration and gene expression in tumour tissue were examined using transcriptome sequencing.

**Results:**

In this cohort, the median follow-up time was 4.5 months, the median progression-free survival was 1.9 months, and the median overall survival (OS) was 4.8 months. The objective response rate was 12.1% and th disease control rate (DCR) was 39.7%. Both the baseline blood neutrophil-to-lymphocyte ratio (bNLR) with a cut-point of 2.7 and the early elevated dynamic change of the bNLR (dNLR) with a cut-point of 1.5 were prognostic factors of survival. Patients in the high bNLR or dNLR group had remarkably poor DCR (25.8% vs 59.1%, *P* = 0.023; 15.8% vs 54.6%, *P* = 0.008). In multivariate analysis, bNLR and tumour mutational burden were independent prognostic factors of OS. Tumour RNA-seq analysis revealed enriched neutrophil infiltration and a higher tumour NLR in the bNLR-high group. Corresponding tumour gene-expression profiles were associated with neutrophil recruitment and inflammatory cytokine aggregation.

**Conclusions:**

Our study demonstrated the potential clinical utility of NLR as a biomarker for patient selection and clinical management in predicting the prognosis of AGC patients as well as response to anti-PD-1 therapy. In addition, high bNLR reflected the imbalance of tumour-tissue-infiltrating neutrophils and lymphocytes, and was associated with an immunosuppressive and pro-tumour microenvironment.

## Introduction

Gastric cancer (GC) is the fifth most common cancer worldwide, with a particularly high incidence in Eastern Asia [1]. Despite significant improvements in survival over the past several decades due to the development of chemotherapy and molecular targeted therapies, the prognosis of advanced gastric cancer (AGC) has remained poor. Recent breakthroughs from immune checkpoint inhibitors (ICIs) have paved the way to a new era of cancer therapy. Anti-programmed death 1 (anti-PD-1) monoclonal antibody was approved as a standard option for patients who failed to respond to second-line or more systematic treatment worldwide [2, 3]. However, because of the limited efficacy and potential severe toxicities of ICIs, immunotherapy cannot be used to treat all AGC patients. Therefore, identifying predictive biomarkers to identify AGC patients who are likely to benefit from anti-PD-1 antibody is critical.

The USA Food and Drug Administration approved pembrolizumab for chemorefractory patients with programmed death ligand 1 (PD-L1)-positive AGC and mismatch repair deficiency or high tumour mutational burden (TMB) (≥10 mutations/Mb) [4–6]. EBV positivity [7] as well as circulating tumour DNA and immune-related gene signatures have also been reported as possible biomarkers for ICI efficacy [8]. Increasing attention has been given to blood inflammatory markers including neutrophil count, neutrophil-to-lymphocyte ratio (NLR), and platelet-to-lymphocyte ratio (PLR) [9–12]. Previous studies showed that both baseline NLR and derived NLR have a predictive value for outcome in advanced melanoma patients treated with nivolumab [9]. Advanced non-small cell lung cancer (NSCLC) patients treated with PD-1/PD-L1 inhibitors who had lower baseline NLR had better response and longer survival than those who had higher baseline NLR [10, 13, 14]. However, uncertainty exists around blood inflammatory biomarker associations with GC immunotherapy outcome.

This study was designed to comprehensively evaluate the predictive and prognostic value of blood inflammatory biomarkers based on the analysis of clinicopathological characteristics, treatment outcomes, haematological parameters, and tumour transcriptome evaluation by RNA sequencing (RNA-seq) data. In this study, we characterized gene-expression patterns in tumours using RNA-seq and compared different tumour microenvironment (TME) characteristics in different NLR groups to further explore the association between NLR and the TME. The aim of these analyses was to evaluate the strength and validity of evidence on the association between NLR and the prognosis of AGC patients treated with anti-PD-1 antibody.

## Patients and methods

### Patients

We retrospectively analysed the data of 58 patients in cohort 1 from a prospective, multi-centre phase Ib/II study (ClinicalTrials.gov identifier: NCT 02915432) [15] investigating the safety and efficacy of the anti-PD-1 antibody toripalimab in chemorefractory AGC or gastro-oesophageal junction adenocarcinoma and the predictive survival benefit of TMB and PD-L1. Toripalimab was given at 3 mg/kg once every 2 weeks. The following data were collected: age, sex, Eastern Cooperative Oncology Group Performance Status (ECOG PS), body mass index (BMI), previous treatment, objective response rate (ORR), disease control rate (DCR), progression-free survival (PFS), overall survival (OS), adverse events (AEs), tumour PD-L1 expression, and TMB. This study was approved by the Institutional Review Board and Ethics Committee of Sun Yat-sen University Cancer Center (approval number: B2020-152–01) and was conducted in accordance with the Helsinki Declaration and the international standards of good clinical practice.

### Haematological parameters

Blood-test results, including white blood cell (WBC) count, absolute lymphocyte count (ALC), absolute neutrophil count (ANC), absolute monocyte count (AMC), and platelet (PLT) count, were recorded at baseline and 2 weeks after the first administration of toripalimab. Blood NLR (bNLR) was calculated as ANC/ALC, PLR as PLT/ALC, and lymphocyte-to-monocyte ratio (LMR) as ALC/AMC. Dynamic change of bNLR (dNLR) was calculated as bNLR at 2 weeks/bNLR baseline. The cut-off points for NLR, PLR, and LMR were determined by the Youden’s index using receiver-operating characteristic (ROC) analysis.

### Tumour evaluation

Tumour response assessments according to Response Evaluation Criteria in Solid Tumors version 1.1 were performed every 8 weeks during the first year of treatment and then every 12 weeks until disease progression or therapy discontinuation. AEs were graded according to the National Cancer Institute Common Terminology Criteria version 4.0.

PD-L1 expression was detected by immunohistochemistry (IHC) staining with the anti-human PD-L1 monoclonal antibody SP142. PD-L1-positive status was defined as membrane staining in ≥1% of tumour cells or the presence of PD-L1 staining of any intensity in tumour-infiltrating immune cells (ICs). TMB was detected by whole-exome sequencing on tumour biopsies and determined by analysing somatic mutations per mega-base (Mb). As previously reported, a cut-off of the top 20% of the TMB (12 mutations/Mb) was selected for defining a tumour as TMB-high (TMB-H). Patients with a TMB of <12 mutations/Mb were defined as TMB-low (TMB-L) [15].

### Tumour-tissue transcriptome sequencing

Tumour-tissue transcriptional profiling was performed to characterize the molecular phenotypes using RNA-seq. The raw reads were processed and aligned to the UCSC hg 38 reference genome using STAR and then quantified by RSEM [16, 17]. Gene expression is presented as transcripts per million values. Gene set enrichment analysis (GSEA) within the HALLMARK gene set database was performed with GSEA v4.0.3 for Windows (https://www.gsea-msigdb.org/gsea/downloads.jsp). Immune-related genes and their functional classifications were obtained from Thorsson *et al.* [18]. Neutrophil-related genes were described in a previous study [19]. The R package microenvironment cell population-counter (MCP-counter) [20] was used to estimate the abundance of tumour-infiltrating leukocytes. Tumour NLR (tNLR) was calculated as neutrophils/(T-cells + B-cells).

RNA-seq data of melanoma, glioblastoma, and urothelial cancer patients who received ICIs from four published studies [21–24] were collected and analysed for validation purposes. The median value of tNLR was used as the cut-off point, and patients were divided into the tNLR-high group and the tNLR-low group using this cut-off value.

### Statistical analysis

Patient characteristics were summarized using descriptive statistics. OS was calculated from the date of the first treatment to either death or the last follow-up date; PFS was calculated from the date of the first treatment to the date of disease progression, death, or last follow-up. The cut-off points were determined by Youden’s index using ROC analysis. Comparisons of clinicopathological characteristics in different bNLR groups were performed using Pearson’s chi-square test. Univariate analyses and multivariate analyses of variables for OS and PFS were performed using a Cox’s proportional hazards model. Comparisons of ORR, DCR, and AEs were performed by Pearson’s chi-square test. The concordance index (C-index) was used to estimate the predictive capacity. A two-tailed Student’s *t*-test was used to analyse the differences between tNLR groups. A value of *P* < 0.05 was considered significant. Statistical analyses were performed using IBM-SPSS version 23.0, GraphPad Prism 8 software, or R software version 3.6.1.

## Results

### Patient characteristics and treatment outcomes

A total of 58 stage chemorefractory AGC patients were included in this study between December 2016 and September 2017. The baseline patient characteristics are summarized in Table 1. The median age of patients was 60 years (range, 52–66 years) and 70.7% of patients were male. Forty-five (77.6%) patients had previously received at least two lines of systemic treatments. Among the 55 samples with valid PD-L1 IHC staining results, 8 tumours were PD-L1-positive. TMB results were available for 54 patients and 12 tumours were defined as TMB-H. The median follow-up time was 4.5 months, the median PFS of the overall patient group was 1.9 months, and the median OS was 4.8 months. The ORR was 12.1% (7/58) and the DCR was 39.7% (23/58), including 7 patients with partial response (PR) and 16 with stable disease (SD). Forty-five (77.6%) patients experienced at least one treatment-related AE (TRAE) of any grade and 13 (22.4%) patients experienced at least one grade 3 or higher TRAE. Fifteen (25.9%) patients experienced immune-related AEs (irAEs).

**Table 1.** Baseline characteristics and haematological parameters of 58 AGC patients in this study

Characteristic	Median (range) or number of cases (%)
Age (years)	60 (52–66)
Sex (male/female)	41 (70.7)/17 (29.3)
ECOG performance status	
0	20 (34.5)
1	38 (65.5)
BMI	
≥25	9 (15.5)
<25	49 (84.5)
Prior lines	
1L	13 (22.4)
2L+	45 (77.6)
PD-L1^a^	
Positive	8 (13.8)
Negative	47 (81.0)
NA	3 (5.2)
TMB	
≥12 mutations/Mb	12 (20.7)
<12 mutations/Mb	42 (72.4)
NA	4 (6.9)
ANC (10^9^/L)	4.4 (1.7–8.4)
ALC (10^9^/L)	1.3 (0.5–2.5)
NLR	
>2.7	31 (53.5)
≤2.7	22 (37.9)
NA	5 (8.6)
PLR	
>267	11 (19.0)
≤267	42 (72.4)
NA	5 (8.6)
LMR	
>2.8	21 (36.2)
≤2.8	32 (55.2)
NA	5 (8.6)

ECOG, Eastern Cooperative Oncology Group; BMI, body mass index; TMB, tumour mutational burden; NA, not available; ANC, absolute neutrophil count; ALC, absolute lymphocyte count; NLR, neutrophil-to-lymphocyte ratio; PLR, platelet-to-lymphocyte ratio; LMR, lymphocyte-to-monocyte ratio.

^a^Positive defined as ≥1% of tumour cells or immune cells showing IHC staining for PD-L1 using SP142 antibody .

### Relation between bNLR and treatment outcomes

**Figure 1.**
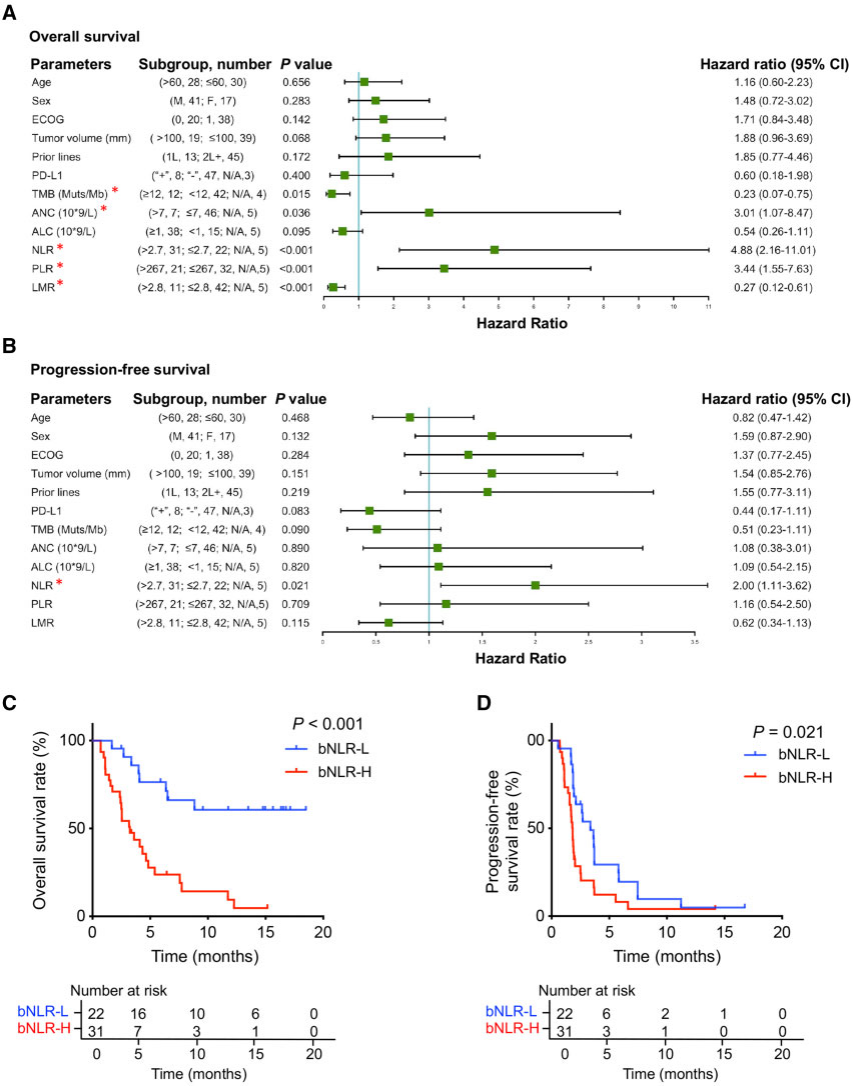
Risk factors and survival curves of advanced gastric cancer patients treated with the anti-PD-1 monoclonal antibody toripalimab. (A) and (B) Forest plot of univariate analysis for risk factors associated with overall survival (OS) and progression-free survival (PFS). (C) and (D) OS and PFS in patients stratified by the baseline neutrophil-to-lymphocyte ratio (bNLR) level: bNLR-L (≤2.7) and bNLR-H (>2.7). *P < 0.05.

Fifty-three patients had detailed baseline blood-test data, including ALC, ANC, and AMC levels. We defined the best cut-off points as 2.7 for bNLR, 267 for PLR, and 2.8 for LMR according to ROC analysis. Univariate analysis showed that bNLR, TMB, ANC, PLR, LMR, and TMB had statistically significant impacts on OS, but bNLR was the only prognostic factor for PFS (Figure 1A–D). Multivariate analysis showed that bNLR was an independent prognostic factor for both PFS and OS, while only TMB significantly impacted OS (Table 2). There was no significant difference in ORR between the bNLR-low group and bNLR-high group (13.6% vs 6.5%, *P* = 0.638). The bNLR-low group had a significantly higher DCR than the bNLR-high group (59.1% vs 25.8%, *P* = 0.023) (Table 3). There were no significant differences in clinicopathological characteristics between the bNLR-low group and the bNLR-high group (Table 4).

**Table 2.** Multivariate analyses of factors associated with overall survival and progression-free survival

Parameter	Overall survival		Progression-free survival	
	Hazard ratio (95% CI)	*P*-value	Hazard ratio (95% CI)	*P*-value
TMB	0.17 (0.47–0.59)	0.005^*^	0.53 (0.23–1.19)	0.124
PD-L1	–	–	0.54 (0.21–1.42)	0.214
ANC	2.89 (0.86–9.62)	0.085	–	–
NLR	11.41 (1.98–65.76)	0.006^*^	2.14 (1.17–3.90)	0.013^*^
PLR	2.22 (0.82–6.05)	0.119	–	–
LMR	2.14 (0.39–11.69)	0.380	–	–

TMB, tumour mutational burden; ANC, absolute neutrophil count; NLR, neutrophil-to-lymphocyte ratio; PLR, platelet-to-lymphocyte ratio; LMR, lymphocyte-to-monocyte ratio; CI, confidence interval.

* *P* < 0.05.

**Table 3.** Comparison of clinical efficacy in different NLR groups

Group	*n* ^a^	PR, *n*	SD, *n*	ORR, *n* (%)	DCR, *n* (%)
bNLR					
≤2.7	22	3	10	3 (13.6)	13 (59.1)
>2.7	31	2	6	2 (6.5)	8 (25.8)
*P*-value^b^				0.638	0.023^*^
dNLR					
≤1.5	33	4	14	4 (12.1)	18 (54.6)
>1.5	19	1	2	1 (5.3)	3 (15.8)
*P*-value				0.641	0.008^*^
bNLR, dNLR					
≤2.7, ≤1.5	13	2	9	2 (15.4)	11 (84.6)
>2.7, ≤1.5	20	2	5	2 (10.0)	7 (35.0)
≤2.7, >1.5	8	1	1	1 (12.5)	3 (25.0)
>2.7, >1.5	11	0	1	0 (0)	1 (9.1)
*P*-value				0.685	0.001^*^

PR, partial response; SD, stable disease; ORR, objective response rate; DCR, disease control rate; bNLR, baseline blood neutrophil-to-lymphocyte ratio; dNLR, dynamic change of blood NLR.

^a^Patients with detailed bNLR data (*n* = 53) and detailed dNLR data (*n* = 52) were included in the analyses.

^a^Pearson’s Chi-square test, *P < 0.05.

**Table 4.** Baseline characteristics of 53 AGC patients according to different bNLR levels^a^

Characteristic	bNLR-high (>2.7)	bNLR-low (≤2.7)	*P*-value^c^
Age (years)			
<60	14 (45.2)	12 (54.5)	0.501
≥60	17 (54.8)	10 (45.5)	
Sex			
Male	21 (67.7)	17 (77.3)	0.448
Female	10 (32.3)	5 (22.7)	
ECOG performance status			
0	9 (29.0)	7 (31.8)	0.828
1	22 (71.0)	15 (68.2)	
BMI			
≥25	11 (35.5)	8 (36.4)	0.948
<25	20 (64.5)	14 (63.6)	
Prior lines			
1L	7 (22.6)	3 (13.6)	0.412
2L+	24 (77.4)	19 (86.4)	
PD-L1^b^			
Positive	3 (9.7)	3 (13.6)	0.689
Negative	27 (87.1)	19 (86.4)	
NA	1 (3.2)		
TMB			
≥12 mutations/Mb	7 (22.6)	4 (18.2)	0.780
<12 mutations/Mb	23 (74.2)	16 (72.7)	
NA	1 (3.2)	2 (9.1)	

The values are presented as the number of cases following the percentage in parentheses.

ECOG, Eastern Cooperative Oncology Group; BMI, body mass index; TMB, tumour mutational burden; NA, not available; NLR, neutrophil-to-lymphocyte ratio.

^a^Patients with detailed bNLR data (*n* = 53) were included in the analyses.

^b^Positive is defined as ≥1% of tumour cells or immune cells showing IHC staining for PD-L1 with SP142 antibody.

^c^Pearson’s Chi-square test.

### Relation between dNLR and treatment outcomes

**Figure 2.**
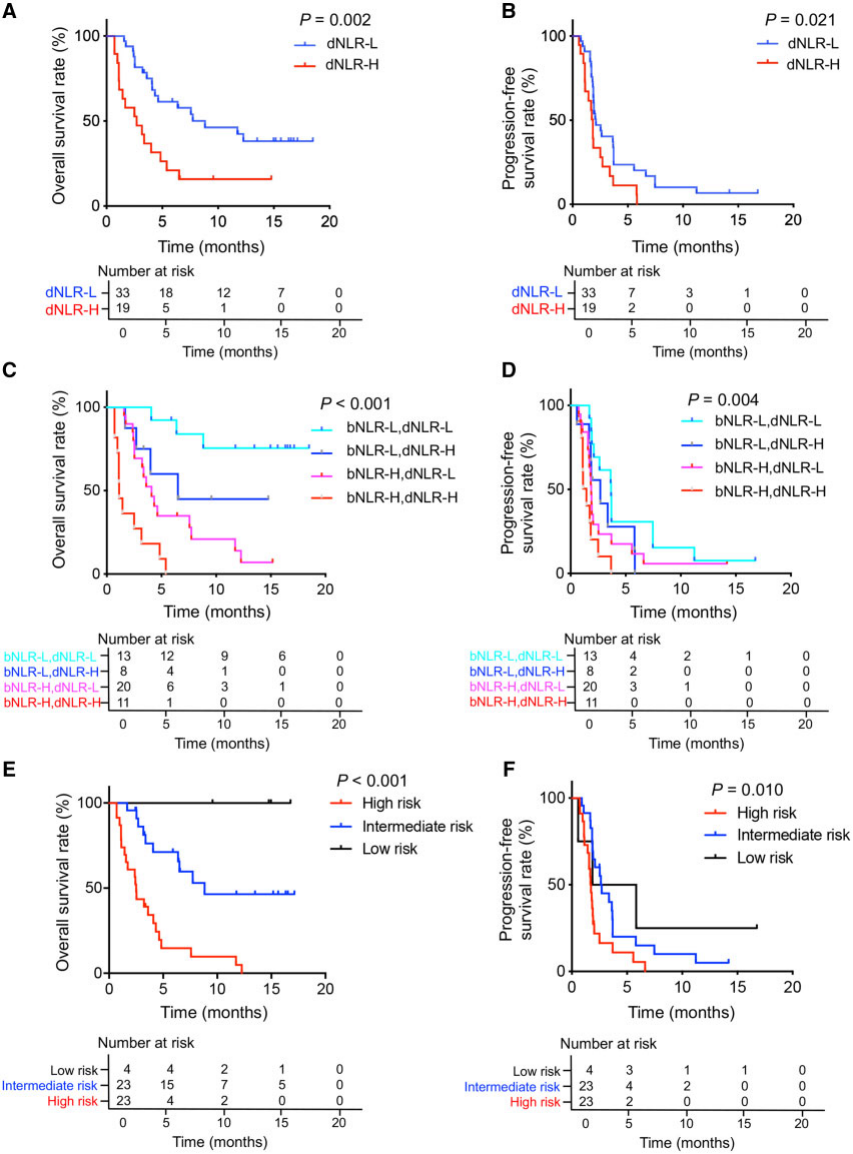
Survival curves of advanced gastric cancer patients treated with toripalimab. (A) and (B) OS and PFS of patients stratified by the early dynamic change of NLR (dNLR) after the first dose of toripalimab. We defined dNLR-L as dNLR ≤1.5 and dNLR-H as dNLR >1.5. (C) and (D) OS and PFS of patients stratified according to the combination of bNLR (bNLR-L ≤2.7, bNLR-H >2.7) and dNLR (dNLR-L ≤1.5, dNLR-H >1.5). (E) and (F) OS and PFS of patients stratified according to the combination of NLR and TMB. One point was assigned for a negative factor (TMB <12 mutations/Mb or NLR >2.7). Patients were scored and classified into the high-risk group (2 points); intermediate-risk group (1 point); or low-risk group (0 points).

Significant survival differences were observed between patients with elevated dNLR and those without elevated dNLR after the first dose of toripalimab. There were 52 patients with detailed dNLR data. According to the ROC analysis, the best cut-off value of dNLR was 1.5. We divided the patients into the dNLR-high (>1.5) group (*n* = 19) and the dNLR-low (≤1.5) group (*n* = 33). The dNLR-low group had longer median PFS (2 vs 1.7 months, *P* = 0.021) and median OS (6.4 vs 2.6 months, *P* = 0.002) than the dNLR-high group (Figure 2A and B). No difference in ORR was observed between the groups (12.1% vs 5.3%, *P* = 0.641). The dNLR-low group had a significantly higher DCR (54.4% vs 15.8%, *P* = 0.008) (Table 3). In the evaluation of patients according to both baseline bNLR and dNLR, patients with a bNLR^Low^/dNLR^Low^ status had the highest DCR (84.6%), while those with bNLR^High^/dNLR^High^ status had the lowest DCR (9.1%) (Table 3). Similar results were observed in the survival analysis. Patients with bNLR^Low^/dNLR^Low^ status had a significantly better survival outcome than those with bNLR^High^/dNLR^High^ status (median OS: 14.3 vs 1.1 months, *P* < 0.001; median PFS: 3.6 vs 1.1 months, *P* = 0.004) (Figure 2C and D).

### Relation between NLR and AEs

The impacts of baseline bNLR level on TRAEs and irAEs are presented in Table 5. No significant differences in the rates of TRAEs and irAEs were found between the bNLR-high group and the bNLR-low group. Interestingly, the dNLR-high group had a lower rate of TRAEs than the dNLR-low group (57.9% vs 87.9%, *P* = 0.031). In the evaluation of patients according to both bNLR and dNLR, patients with bNLR^Low^/dNLR^Low^ status had the highest rate of TRAEs while those with bNLR^High^/dNLR^High^ status had the lowest rate of TRAEs (92.3% vs 63.6%, respectively).

**Table 5.** Treatment-related adverse events (TRAEs) and immune-related adverse events (irAEs) in different NLR groups

Group	TRAEs	TRAEs grade ≥3	irAEs
bNLR			
≤2.7	17 (77.3)	4 (18.2)	7 (31.2)
>2.7	23 (74.2)	8 (25.8)	7 (22.6)
*P*-value	0.797	0.740	0.452
dNLR			
≤1.5	28 (87.9)	7 (21.2)	10 (30.3)
>1.5	11 (57.9)	5 (26.3)	4 (21.1)
*P*-value	0.031^*^	0.471	0.534
Combination of bNLR and dNLR			
≤2.7, ≤1.5	12 (92.3)	3 (23.1)	5 (38.5)
>2.7, ≤1.5	15 (75.0)	4 (20.0)	5 (25.0)
≤2.7, >1.5	6 (75.0)	1 (12.5)	2 (25.0)
>2.7, >1.5	7 (63.6)	4 (36.4)	2 (18.2)
*P*-value	0.422	0.686	0.757

The values are presented as the number of cases following the percentage in parentheses.

TRAEs, treatment-related adverse events; irAEs, immune-related adverse events; bNLR, baseline blood neutrophil-to-lymphocyte ratio; dNLR, dynamic change of blood NLR.

* *P* < 0.05.

### Combination of bNLR and TMB for prognostic prediction

In our study, baseline bNLR and TMB were independent prognostic factors for OS benefit from anti-PD-1 antibody treatment. We developed a combination scoring system in which one point was assigned for each negative prognostic factor (TMB <12 mutations/Mb or bNLR >2.7) in each patient. Fifty patients with both TMB and bNLR data were included and they were classified into three groups based on the combination score: the high-risk group (2 points, *n* = 23), the intermediate-risk group (1 point, *n* = 23), and the low-risk group (0 points, *n* = 4). The median OS of the patients in the high-, intermediate-, and low-risk groups was 2.5, 6.4, and 14.9 months, respectively [hazard ratio (HR): 4.87, 95% confidence interval (CI) 2.441–9.719, *P* < 0.001], whereas the median PFS was 1.7, 2.5, and 3.9 months, respectively (HR: 1.97, 95% CI 1.174–3.295, *P* = 0.010) (Figure 2E and F). The C-index value of the combined score for OS was 0.727 (95% CI 0.657–0.798), while the C-index values of bNLR and TMB were 0.676 (95% CI 0.607–0.746) and 0.606 (95% CI 0.539–0.673), respectively.

### Tumour RNA-seq analysis in different bNLR groups

**Figure 3.**
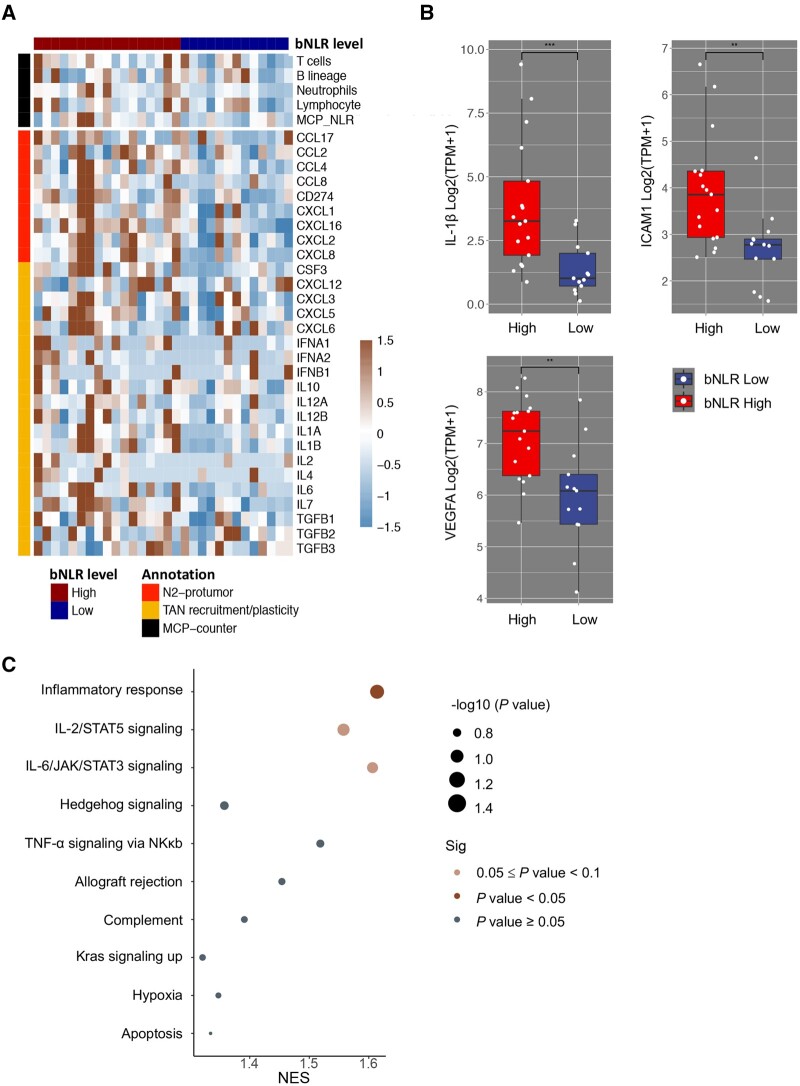
RNA-seq analysis of tumour tissue in different NLR groups. (A) Heat map of tumour infiltrated neutrophil and lymphocyte densities (black colour) and differential expression of neutrophil-associated genes (red and yellow colour) between the bNLR-high group (>2.7) and the bNLR-low group (≤2.7). (B) The top three significantly different immune-related genes in the bNLR-high group were IL-1β, ICAM1, and VEGFA genes. The full gene lists are shown in Supplementary Table 2. (C) Representative enriched signalling pathways of the bNLR-high group. Enrichment analysis was performed using the HALLMARK gene set database. *P < 0.05; **P < 0.01; ***P < 0.001.

In our study, 51.7% (30/58) of patients had baseline tumour samples that were available for transcriptome sequencing. Using the cut-off value of 2.7 of baseline bNLR, the 30 patients were divided into the bNLR-high group (>2.7, *n* = 17) and the bNLR-low group (≤2.7, *n* = 13). MCP-counter was applied to compare the tumour-infiltrating ICs between the two groups. In the bNLR-high group, neutrophils were the most significantly enriched cells in tumour tissues (Supplementary Table 1) and tNLR was significantly increased (average MCP NLR, 0.83 vs 0.55, *P* = 0.050). We did not observe any lymphocyte depletion. Next, we analysed the immune-related differentially expressed genes (DEGs) and neutrophil-related DEGs, and found that the biomarkers related to neutrophil recruitment and tumour-associated neutrophil (TAN) plasticity were significantly enriched in the bNLR-high group compared with the bNLR-low group (Figure 3A). The top three significantly different immune-related genes in the NLR-high group were IL-1β, ICAM1, and VEGFA (Figure 3B). The full gene lists are shown in Supplementary Table 2 and Table 3. Finally, we conducted GSEA to investigate the biological pathways enriched in the bNLR-high group. The top-scoring gene sets (FDR < 0.1) were inflammatory response signalling, IL-2/STAT5 signalling, and IL-6/JAK/STAT3 signalling (Figure 3C).

### Validated analysis of tumour NLR in different cancer cohorts from published studies

**Figure 4.**
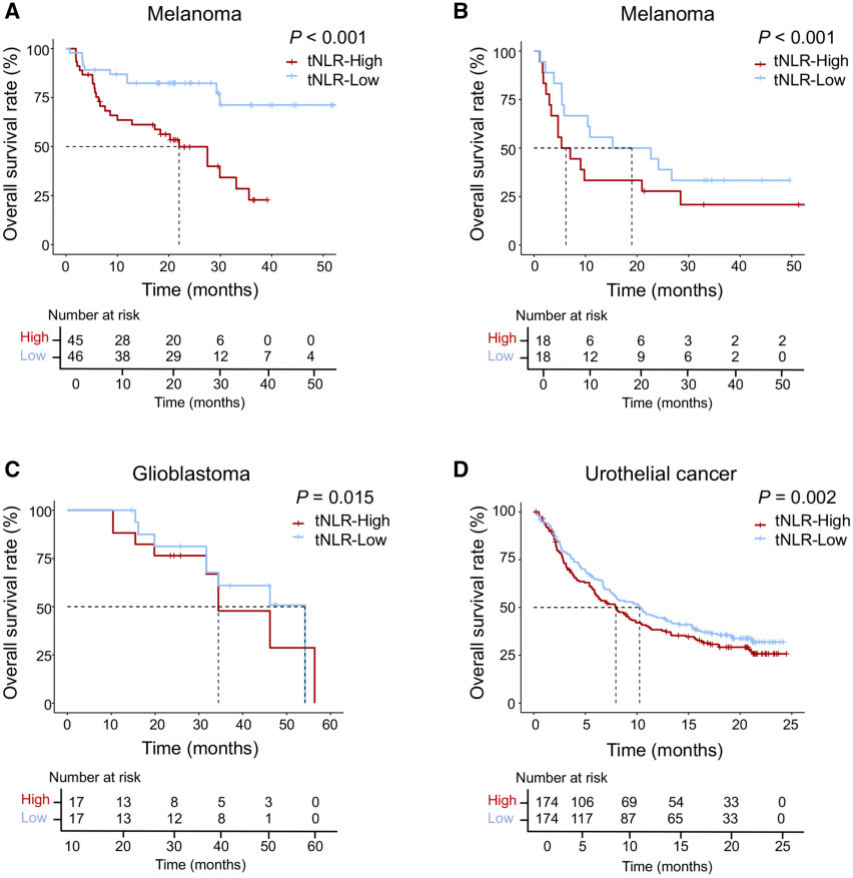
Tissue NLR (tNLR) and prognosis in four cohorts of cancer patients. Patients were divided into the tNLR-high and tNLR-low groups using the median tNLR value as the cut-off point. The OS of patients treated with ICI treatment is stratified according to tNLR in: (A) and (B) the melanoma cohort; (C) the glioblastoma cohort; and (D) the urothelial cancer cohort.

The results of this study indicate that elevated bNLR was related to poor survival of GC patients receiving anti-PD-1 antibody treatment and indirectly reflected the neutrophil–lymphocyte imbalance in tumour tissue. We further collected and analysed four published independent gene-expression datasets of melanoma, glioblastoma, and urothelial cancer patients who received ICI treatment. We used the MCP-counter to estimate the abundance of tumour-infiltrating leukocytes and defined the median tNLR value as the cut-off point. A significant increase in OS benefit was observed in the tNLR-low group in each cohort (Figure 4A–D).

## Discussion

As ICIs have become a promising option for the treatment of AGC patients, there has been increasing recognition that simple predictive biomarkers for the selection of patients who might benefit from ICIs are needed. In recent years, blood inflammatory markers have been explored as potential biomarkers, because they might reflect the inflammatory response to cancer. WBC count and C-reactive protein are the most commonly used biochemical parameters to evaluate the systemic inflammatory response. Platelets are also involved in the systemic inflammatory response, as thrombocytopenia is a common phenomenon in cancer patients [25]. In addition, NLR, PLR, and LMR have been strongly associated with prognosis in several types of cancer [26–28]. Our study demonstrated the predictive and prognostic value of bNLR, dNLR, and tNLR based on the analysis of clinicopathological characteristics, haematological parameters, and tumour RNA-seq data from a prospective clinical trial that investigated the safety and efficacy of anti-PD-1 monotherapy in chemorefractory AGC patients. Furthermore, we explored different TME characteristics in different bNLR groups by comparing their tumour-gene-expression profiles.

In this study, in addition to the high baseline bNLR, an elevated dNLR at 2 weeks after one dose of toripalimab was also significantly associated with poor prognosis and low response. According to the ROC analysis, the best cut-off value of dNLR was 1.5. The dNLR-low patient group (≤1.5) had longer median PFS and median OS than the dNLR-high group. No difference in ORR was observed between the two groups, but the dNLR-low group had a significantly higher DCR. Upon evaluation of patients based on a combination of baseline bNLR and dNLR, patients with a bNLR^Low^/dNLR^Low^ status had the highest DCR, while those with bNLR^High^/dNLR^High^ status had the lowest DCR. Similar results were observed in the survival analysis. The patients with a bNLR^Low^/dNLR^Low^ status had significantly improved survival outcomes than those with a bNLR^High^/dNLR^High^ status.

These findings support the idea that baseline bNLR and dynamic monitoring of bNLR may help clinicians to identify patients who benefit from ICIs. However, the optimal cut-off point of NLR still needs to be defined, since various findings have been reported for different types of cancer. In our study, we defined 2.7 as the cut-off value for baseline bNLR and 1.5 as the cut-off value for dNLR by analysing the ROC curve. Ota *et al*. [29] reported that a baseline NLR of >3 was associated with poor survival for AGC patients with nivolumab monotherapy. In melanoma and NSCLC, the most common cut-off value is an NLR of ≥5 [9]. In a meta-analysis that included 10 studies with 2,952 cases of GC, the selected cut-off values ranged from 1.44 to 5.0, based on the ROC curve, median value, or reported studies [30]. The results suggested that an elevated NLR could predict poor OS regardless of the sample size and cut-off values. Despite the uncertainty of baseline NLR values, our study demonstrated that the early dynamic change of NLR after ICI therapy could be regarded as an effective predictor of immunotherapy outcome.

In our phase Ib/II study, TMB-H (>12 mutations/Mb) was recognized as a prognostic factor for OS [15]. We combined the baseline bNLR with TMB to classify patients into different risk groups. The median OS in the high-risk group and low-risk group were 2.5 and 14.9 months, respectively. The combination of markers demonstrated a higher C-index for OS than either bNLR or TMB alone. These results may explain the limitations of a single tumour-associated biomarker in predicting the prognosis of immunotherapies, even in patients with high TMB. Indeed, systemic inflammatory status may play an important role in the response to ICI treatments. Our findings support using the combination of bNLR and TMB for identifying candidates likely to receive the most or least benefit from ICI treatment.

In the tumour tissue of bNLR-high patients, we observed enriched neutrophils and monocytic lineage cell infiltration as well as a higher tNLR. These results revealed that bNLR, as a systemic inflammatory marker, was related to the tumour inflammatory status, which was mostly manifested by TAN infiltration and inflammatory cytokine aggregation. We also showed that a higher tNLR was significantly associated with poor prognosis in four independent cohorts of melanoma, glioblastoma, and urothelial cancer patients treated with ICIs. Gene-expression profiling of corresponding tumour tissue further provided some evidence on the mechanisms underlying the association between inflammation and immunotherapy responses. Cytokines and chemokines related to neutrophil recruitment or TAN functional plasticity were highly enriched and angiogenesis-associated genes (VEGFA, ICAM1, and MMP9 genes) and cytokine molecules (CXCL1, IL-8, and IL-1β) were significantly overexpressed. Previous studies showed that these neutrophil-derived molecules are essential for tumour angiogenesis [31]. GSEA displayed gene enrichment in the inflammatory response as well as in IL-2/STAT5 and IL-6/JAK/STAT3 signalling. A previous study reported that activation of the STAT3 signalling increased immunosuppressive cytokine and mediator expression [32]. These results show that cancer progression and inflammatory conditions share common regulatory pathways and molecules, such as pro-inflammatory cytokines and pro-angiogenic factors. Adding to these findings, our study provided evidence that peripheral bNLR is an indicator of more permissive TME and impaired antitumour immunity, and this further suggests that adding neutrophil antagonist or angiogenesis inhibitors might be a feasible strategy for the bNLR-H group to benefit from ICI treatments.

NLR has not been widely used for clinical application as an immunotherapy prognostic and and/or predictive marker. NLR can be affected by acute infection and severe treatment-related factors such as chemotherapy or the use of granulocyte colony stimulating factors. Although bNLR has been reported as a simplified surrogate predictive marker for patients treated with ICIs in several types of cancer, the biological basis for this effect remains to be determined. Previous studies provided some possible explanations for these findings. For instance, elevated bNLR was attributed to neutrophilia or lymphopenia. In addition, neutrophilia is an active, intra-tumoural source of many cytokines and chemokines that play important roles in tumour development and the immunosuppressive network [33]. The lymphocyte-depleted environment also contributed to poor response to ICIs [34, 35]. Kargl *et al.* [36] reported that CD8+ T-cells and neutrophils were inversely associated in NSCLC tumour tissues, and their ratio could predict the outcome of anti-PD-1 monotherapy.

There are several limitations to our study. First, the number of patients is small and a validation cohort in larger prospective studies is needed. Second, we assessed tumour-tissue immune-cell infiltrations by RNA-seq analysis and this accuracy is lower than that of directly detecting ICs. Whether bNLR remains predictive for ICI-based combined therapy still needs more evidence from future studies.

## Conclusions

Our study has demonstrated the potential clinical utility of baseline bNLR and dNLR as easy-to-use biomarkers to predict the response and prognosis of chemorefractory AGC patients who received anti-PD-1 antibody therapy. These findings might be useful to improve patient selection and clinical management. In addition, the gene-expression profiling of tumour tissue revealed that high bNLR reflected the imbalance of tumour-tissue-infiltrating neutrophils and lymphocytes, and was associated with an immunosuppressive and pro-tumour microenvironment .

## Supplementary Data

Supplementary data is available at *Gastroenterology Report* online.

## Authors’ Contributions

(i) Conception and design: F.H.W., S.Q.Y., D.Y.R., and Y.X.C.; (ii) administrative support: F.H.W. and S.Q.Y.; (iii) provision of study materials or patients: F.H.W., S.Q.Y., and R.H.X.; (iv) collection and assembly of data: D.Y.R., X.L.W., and Y.N.W.; (v) data analysis and interpretation: Y.X.C. and Z.X.W.; (vi) manuscript writing: all authors; (vii) final approval of the manuscript: all authors. D.Y.R. and Y.X.C. contributed equally to this study.

## Funding

This study was supported by the CAMS Innovation Fund for Medical Sciences (CIFMS) [2019-I2M-5–036] and the Science and Technology Program of Guangdong [2019B020227002 ].

## Supplementary Material

goab032_supplementary_data

## References

[1] Sung H , Ferlay J, Siegel RL et al Global Cancer Statistics 2020: GLOBOCAN estimates of incidence and mortality worldwide for 36 cancers in 185 countries. CA Cancer J Clin 2021;71:209–49. 33538338 10.3322/caac.21660

[2] Kang YK , Boku N, Satoh T et al Nivolumab in patients with advanced gastric or gastro-oesophageal junction cancer refractory to, or intolerant of, at least two previous chemotherapy regimens (ONO-4538-12, ATTRACTION-2): a randomised, double-blind, placebo-controlled, phase 3 trial. Lancet 2017;390:2461–71. 28993052 10.1016/S0140-6736(17)31827-5

[3] Janjigian YY , Bendell J, Calvo E et al CheckMate-032 study: efficacy and safety of nivolumab and nivolumab plus ipilimumab in patients with metastatic esophagogastric cancer. J Clin Oncol 2018;36:2836–44. 30110194 10.1200/JCO.2017.76.6212 PMC6161834

[4] Le DT , Uram JN, Wang H et al PD-1 blockade in tumors with mismatch-repair deficiency. N Engl J Med 2015;372:2509–20. 26028255 10.1056/NEJMoa1500596 PMC4481136

[5] Garon EB , Rizvi NA, Hui R et al; KEYNOTE-001 Investigators. Pembrolizumab for the treatment of non-small-cell lung cancer. N Engl J Med 2015;372:2018–28. 25891174 10.1056/NEJMoa1501824

[6] Fuchs CS , Doi T, Jang RW et al Safety and efficacy of pembrolizumab monotherapy in patients with previously treated advanced gastric and gastroesophageal junction cancer: phase 2 clinical KEYNOTE-059 trial. JAMA Oncol 2018;4:e180013. 29543932 10.1001/jamaoncol.2018.0013 PMC5885175

[7] Kim ST , Cristescu R, Bass AJ et al Comprehensive molecular characterization of clinical responses to PD-1 inhibition in metastatic gastric cancer. Nat Med 2018;24:1449–58. 30013197 10.1038/s41591-018-0101-z

[8] Jin Y , Chen DL, Wang F et al The predicting role of circulating tumor DNA landscape in gastric cancer patients treated with immune checkpoint inhibitors. Mol Cancer 2020;19:154. 33126883 10.1186/s12943-020-01274-7 PMC7596978

[9] Capone M , Giannarelli D, Mallardo D et al Baseline neutrophil-to-lymphocyte ratio (NLR) and derived NLR could predict overall survival in patients with advanced melanoma treated with nivolumab. J Immunother Cancer 2018;6:74. 30012216 10.1186/s40425-018-0383-1 PMC6048712

[10] Diem S , Schmid S, Krapf M et al Neutrophil-to-lymphocyte ratio (NLR) and Platelet-to-lymphocyte ratio (PLR) as prognostic markers in patients with non-small cell lung cancer (NSCLC) treated with nivolumab. Lung Cancer 2017;111:176–81. 28838390 10.1016/j.lungcan.2017.07.024

[11] Kartolo A , Holstead R, Khalid S et al Serum neutrophil-to-lymphocyte ratio and platelet-to-lymphocyte ratio in prognosticating immunotherapy efficacy. Immunotherapy 2020;12:785–98. 32657234 10.2217/imt-2020-0105

[12] Bartlett EK , Flynn JR, Panageas KS et al High neutrophil-to-lymphocyte ratio (NLR) is associated with treatment failure and death in patients who have melanoma treated with PD-1 inhibitor monotherapy. Cancer 2020;126:76–85. 31584709 10.1002/cncr.32506 PMC6906249

[13] Zer A , Sung MR, Walia P et al Correlation of neutrophil to lymphocyte ratio and absolute neutrophil count with outcomes with PD-1 axis inhibitors in patients with advanced non-small-cell lung cancer. Clin Lung Cancer 2018;19:426–34.e1. 29803574 10.1016/j.cllc.2018.04.008

[14] Jiang T , Bai Y, Zhou F et al Clinical value of neutrophil-to-lymphocyte ratio in patients with non-small-cell lung cancer treated with PD-1/PD-L1 inhibitors. Lung Cancer 2019;130:76–83. 30885355 10.1016/j.lungcan.2019.02.009

[15] Wang F , Wei XL, Wang FH et al Safety, efficacy and tumor mutational burden as a biomarker of overall survival benefit in chemo-refractory gastric cancer treated with toripalimab, a PD-1 antibody in phase Ib/II clinical trial NCT02915432. Ann Oncol 2019;30:1479–86. 31236579 10.1093/annonc/mdz197 PMC6771223

[16] Dobin A , Davis CA, Schlesinger F et al STAR: ultrafast universal RNA-seq aligner. Bioinformatics 2013;29:15–21. 23104886 10.1093/bioinformatics/bts635 PMC3530905

[17] Li B , Dewey CN. RSEM: accurate transcript quantification from RNA-seq data with or without a reference genome. BMC Bioinformatics 2011;12:323. 21816040 10.1186/1471-2105-12-323 PMC3163565

[18] Thorsson V , Gibbs DL, Brown SD et al; Cancer Genome Atlas Research Network. The immune landscape of cancer. Immunity 2018;48:812–30.e14. 29628290 10.1016/j.immuni.2018.03.023 PMC5982584

[19] Shaul ME , Fridlender ZG. Neutrophils as active regulators of the immune system in the tumor microenvironment. J Leukoc Biol 2017;102:343–9. 28264904 10.1189/jlb.5MR1216-508R

[20] Becht E , Giraldo NA, Lacroix L et al Estimating the population abundance of tissue-infiltrating immune and stromal cell populations using gene expression. Genome Biol 2016;17:218. 27765066 10.1186/s13059-016-1070-5 PMC5073889

[21] Zhao J , Chen AX, Gartrell RD et al Immune and genomic correlates of response to anti-PD-1 immunotherapy in glioblastoma. Nat Med 2019;25:462–9. 30742119 10.1038/s41591-019-0349-y PMC6810613

[22] Gide TN , Quek C, Menzies AM et al Distinct immune cell populations define response to anti-PD-1 monotherapy and anti-PD-1/anti-CTLA-4 combined therapy. Cancer Cell 2019;35:238–55.e6. 30753825 10.1016/j.ccell.2019.01.003

[23] Łuksza M , Riaz N, Makarov V et al A neoantigen fitness model predicts tumour response to checkpoint blockade immunotherapy. Nature 2017;551:517–20. 29132144 10.1038/nature24473 PMC6137806

[24] Mariathasan S , Turley SJ, Nickles D et al TGFbeta attenuates tumour response to PD-L1 blockade by contributing to exclusion of T cells. Nature 2018;554:544–8. 29443960 10.1038/nature25501 PMC6028240

[25] Franco AT , Corken A, Ware J. Platelets at the interface of thrombosis, inflammation, and cancer. Blood 2015;126:582–8. 26109205 10.1182/blood-2014-08-531582 PMC4520875

[26] Templeton AJ , McNamara MG, Seruga B et al Prognostic role of neutrophil-to-lymphocyte ratio in solid tumors: a systematic review and meta-analysis. J Natl Cancer Inst 2014;106:dju124. 24875653 10.1093/jnci/dju124

[27] Templeton AJ , Ace O, McNamara MG et al Prognostic role of platelet to lymphocyte ratio in solid tumors: a systematic review and meta-analysis. Cancer Epidemiol Biomarkers Prev 2014;23:1204–12. 24793958 10.1158/1055-9965.EPI-14-0146

[28] Tan D , Fu Y, Tong W et al Prognostic significance of lymphocyte to monocyte ratio in colorectal cancer: a meta-analysis. Int J Surg 2018;55:128–38. 29807167 10.1016/j.ijsu.2018.05.030

[29] Ota Y , Takahari D, Suzuki T et al Changes in the neutrophil-to-lymphocyte ratio during nivolumab monotherapy are associated with gastric cancer survival. Cancer Chemother Pharmacol 2020;85:265–72. 31907646 10.1007/s00280-019-04023-w

[30] Zhang X , Zhang W, Feng LJ. Prognostic significance of neutrophil lymphocyte ratio in patients with gastric cancer: a meta-analysis. PLoS One 2014;9:e111906. 25401500 10.1371/journal.pone.0111906 PMC4234250

[31] Ferrara N. VEGF and the quest for tumour angiogenesis factors. Nat Rev Cancer 2002;2:795–803. 12360282 10.1038/nrc909

[32] Yu H , Kortylewski M, Pardoll D. Crosstalk between cancer and immune cells: role of STAT3 in the tumour microenvironment. Nat Rev Immunol 2007;7:41–51. 17186030 10.1038/nri1995

[33] Sionov RV , Fridlender ZG, Granot Z. The multifaceted roles neutrophils play in the tumor microenvironment. Cancer Microenviron 2015;8:125–58. 24895166 10.1007/s12307-014-0147-5 PMC4714999

[34] Binnewies M , Roberts EW, Kersten K et al Understanding the tumor immune microenvironment (TIME) for effective therapy. Nat Med 2018;24:541–50. 29686425 10.1038/s41591-018-0014-x PMC5998822

[35] Jiang P , Gu S, Pan D et al Signatures of T cell dysfunction and exclusion predict cancer immunotherapy response. Nat Med 2018;24:1550–8. 30127393 10.1038/s41591-018-0136-1 PMC6487502

[36] Kargl J , Zhu X, Zhang H et al Neutrophil content predicts lymphocyte depletion and anti-PD1 treatment failure in NSCLC. JCI Insight 2019;4:e130850. 10.1172/jci.insight.130850 PMC6975266 31852845

