# Supplementary material for: Elevated peripheral blood neutrophil-to-lymphocyte ratio is associated with an immunosuppressive tumour microenvironment and decreased benefit of PD-1 antibody in advanced gastric cancer
Source: Gastroenterol Rep (Oxf). 2021 Oct 5;9(6):560–70. doi: 10.1093/gastro/goab032 (PMC8677531; doi:10.1093/gastro/goab032)
Supplement: goab032_supplementary_data [file goab032_supplementary_data.docx]

**Supplementary Tables**

**Table S1.** Tumor tissue infiltrated immune cells calculated by MCP-counter scores in bNLR-high and bNLR-low groups.

| Items | bNLR-high, mean | bNLR-low, mean | *P* value |
| --- | --- | --- | --- |
| Neutrophils | 6.040 | 4.182 | 0.004 |
| Endothelial cells | 4.266 | 3.029 | 0.022 |
| Monocytic lineage | 6.086 | 4.410 | 0.028 |
| NK cells | 0.876 | 0.379 | 0.031 |
| Fibroblasts | 65.276 | 105.709 | 0.123 |
| CD8 T cells | 1.802 | 0.827 | 0.132 |
| T cells | 5.610 | 4.240 | 0.145 |
| Cytotoxic lymphocytes | 2.964 | 2.926 | 0.245 |
| Myeloid dendritic cells | 0.793 | 0.666 | 0.245 |
| Lymphocyte | 9.459 | 9.024 | 0.509 |
| B lineage | 4.024 | 3.700 | 0.967 |

**Table S2.** The immune-related genes expression in bNLR-high and bNLR-low groups.

| Items | bNLR-high, mean | bNLR-low, mean | | *P* value |
| --- | --- | --- | --- | --- |
| IL1B | 8.579 | 1.019 | | < 0.001 |
| ICAM1 | 13.452 | 5.847 | | 0.002 |
| VEGFA | 150.164 | 66.694 | | 0.003 |
| ADORA2A | 3.994 | 2.104 | | 0.010 |
| CD276 | 15.681 | 8.018 | | 0.010 |
| TNFRSF18 | 5.012 | 2.967 | | 0.014 |
| TNFRSF4 | 6.623 | 2.675 | | 0.022 |
| TGFB1 | 22.868 | 18.474 | | 0.025 |
| PRF1 | 4.505 | 2.528 | | 0.025 |
| CD274 | 1.643 | 0.663 | | 0.028 |
| TNFRSF14 | 23.886 | 16.998 | | 0.028 |
| ITGB2 | 12.000 | 8.231 | | 0.035 |
| KIR2DL1 | 0.109 | 0.000 | | 0.038 |
| ICOSLG | 8.396 | 6.285 | | 0.039 |
| IFNG | 0.573 | 0.124 | | 0.039 |
| TNF | 1.828 | 0.488 | | 0.040 |
| TLR4 | 2.405 | 1.576 | | 0.043 |
| LAG3 | 3.067 | 1.963 | | 0.053 |
| IL1A | 0.843 | 0.187 | | 0.053 |
| CD27 | 4.312 | 3.110 | | 0.059 |
| CXCL9 | 2.330 | 1.178 | | 0.059 |
| Items | bNLR-high, mean | bNLR-low, mean | | *P* value |
| HLA-C | 127.917 | 73.284 | | 0.065 |
| SLAMF7 | 4.795 | 2.940 | | 0.072 |
| CD80 | 0.788 | 0.369 | | 0.094 |
| EDNRB | 2.154 | 1.022 | | 0.103 |
| IL2 | 0.104 | 0.000 | | 0.108 |
| IFNA1 | 0.000 | 0.000 | | 0.110 |
| TNFSF9 | 2.433 | 1.715 | | 0.113 |
| PDCD1 | 3.258 | 2.387 | | 0.155 |
| CTLA4 | 1.926 | 1.006 | | 0.157 |
| IL12A | 0.446 | 0.127 | | 0.172 |
| HLA-A | 167.572 | 94.422 | | 0.183 |
| ICOS | 0.483 | 0.393 | | 0.209 |
| HLA-B | 140.484 | 88.409 | | 0.213 |
| IDO1 | 2.771 | 2.494 | | 0.213 |
| TIGIT | 1.569 | 1.340 | | 0.213 |
| BTN3A1 | 7.943 | 9.671 | | 0.229 |
| ENTPD1 | 16.655 | 13.382 | | 0.229 |
| SELP | 0.530 | 0.400 | | 0.245 |
| CD40LG | 1.362 | 1.720 | | 0.263 |
| VEGFB | 14.383 | 12.999 | | 0.300 |
| BTN3A2 | 7.859 | 7.669 | | 0.320 |
| IL2RA | 4.841 | 4.511 | | 0.320 |
| HLA-DRB5 | 0.646 | 1.975 | | 0.335 |
| MICA | 9.120 | 8.169 | | 0.341 |
| HMGB1 | 27.026 | 20.638 | | 0.363 |
| GZMA | 0.745 | 0.673 | | 0.366 |
| VTCN1 | 0.377 | 0.302 | | 0.379 |
| CD70 | 23.015 | 19.407 | | 0.408 |
| CX3CL1 | 3.994 | 3.243 | | 0.408 |
| IFNA2 | 0.097 | 0.000 | | 0.440 |
| IL4 | 0.000 | 0.000 | | 0.470 |
| IL10 | 4.393 | 5.317 | | 0.592 |
| CD28 | 1.750 | 1.925 | | 0.592 |
| PDCD1LG2 | 0.771 | 0.664 | | 0.621 |
| IL13 | 0.282 | 0.021 | | 0.637 |
| KIR2DL3 | 0.251 | 0.147 | | 0.671 |
| CCL5 | 25.540 | 28.850 | | 0.680 |
| HLA-DQA2 | 1.141 | 1.130 | | 0.722 |
| CXCL10 | 0.994 | 0.983 | | 0.754 |
| HLA-DRB1 | 16.373 | 16.334 | | 0.773 |
| ARG1 | 0.328 | 0.477 | | 0.802 |
| HLA-DQB1 | 13.286 | 5.327 | | 0.805 |
| Items | bNLR-high, mean | | bNLR-low, mean | *P* value |
| HLA-DPB1 | 3.600 | 3.987 | | 0.805 |
| HAVCR2 | 8.930 | 8.278 | | 0.805 |
| TNFRSF9 | 8.655 | 9.974 | | 0.805 |
| HLA-DRA | 26.960 | 14.038 | | 0.869 |
| MICB | 6.512 | 6.994 | | 0.869 |
| HLA-DQB2 | 0.656 | 0.557 | | 0.883 |
| BTLA | 0.610 | 0.682 | | 0.902 |
| HLA-DQA1 | 3.616 | 3.876 | | 0.967 |
| CD40 | 3.418 | 4.002 | | 0.967 |
| TNFSF4 | 1.056 | 1.248 | | 0.967 |
| HLA-DPA1 | 11.826 | 8.647 | | 1.000 |
| C10orf54 | 0.000 | 0.000 | | NA |

**Table S3.** The neutrophil-related genes expression in bNLR-high and bNLR-low groups.

| Items | bNLR-high, mean | bNLR-low, mean | *P* value |
| --- | --- | --- | --- |
| IL1B | 8.579 | 1.019 | < 0.001 |
| CXCL8 | 6.770 | 0.913 | < 0.001 |
| CXCL16 | 38.578 | 15.517 | 0.002 |
| ICAM1 | 13.452 | 5.847 | 0.002 |
| VEGFA | 150.164 | 66.694 | 0.003 |
| IL6 | 1.743 | 0.716 | 0.003 |
| CSF3 | 3.824 | 0.639 | 0.008 |
| CXCL1 | 13.522 | 3.039 | 0.010 |
| IL7 | 5.226 | 2.054 | 0.012 |
| TGFB1 | 22.868 | 18.474 | 0.025 |
| CCL4 | 3.581 | 1.369 | 0.028 |
| CD274 | 1.643 | 0.663 | 0.028 |
| CXCL2 | 14.046 | 5.850 | 0.028 |
| CXCL5 | 12.450 | 4.358 | 0.028 |
| TNF | 1.828 | 0.488 | 0.040 |
| MMP9 | 5.189 | 2.192 | 0.043 |
| IL1A | 0.843 | 0.187 | 0.053 |
| CCL8 | 0.414 | 0.115 | 0.095 |
| IL2 | 0.104 | 0.000 | 0.108 |
| IFNA1 | 0.000 | 0.000 | 0.110 |
| IL12B | 3.685 | 2.636 | 0.157 |
| CCL3 | 2.412 | 1.464 | 0.170 |
| IL12A | 0.446 | 0.127 | 0.172 |
| CXCL3 | 8.354 | 2.284 | 0.198 |
| CXCR4 | 11.889 | 10.535 | 0.213 |
| Items | bNLR-high, mean | bNLR-low, mean | *P* value |
| CCL2 | 4.749 | 3.356 | 0.241 |
| CCL17 | 0.520 | 0.121 | 0.382 |
| IFNA2 | 0.097 | 0.000 | 0.440 |
| IL4 | 0.000 | 0.000 | 0.467 |
| IL10 | 4.393 | 5.317 | 0.592 |
| ELANE | 0.305 | 0.262 | 0.597 |
| ARG1 | 0.328 | 0.477 | 0.802 |
| CXCL6 | 0.326 | 0.503 | 0.867 |
| TGFB2 | 1.521 | 2.605 | 0.869 |
| TGFB3 | 4.124 | 5.149 | 0.869 |
| IFNB1 | 0.000 | 0.000 | 0.940 |
| CXCL12 | 8.229 | 8.110 | 0.967 |
